# Induction of amyloid-β deposits from serially transmitted, histologically silent, Aβ seeds issued from human brains

**DOI:** 10.1186/s40478-020-01081-7

**Published:** 2020-11-30

**Authors:** Anne-Sophie Hérard, Fanny Petit, Charlotte Gary, Martine Guillermier, Susana Boluda, Clément M. Garin, The Brainbank Neuro-CEB Neuropathology Network, Franck Letournel, Marie-Laure Martin-Négrier, Maxime Faisant, Catherine Godfraind, Claude-Alain Maurage, Vincent Deramecourt, Mathilde Duchesne, David Meyronnet, André Maues de Paula, Valérie Rigau, Fanny Vandenbos-Burel, Charles Duyckaerts, Danielle Seilhean, Susana Boluda, Isabelle Plu, Serge Milin, Dan Christian Chiforeanu, Annie Laquerrière, Béatrice Lannes, Suzanne Lam, Marc Dhenain

**Affiliations:** 1 Laboratoire des Maladies Neurodégénératives, Université Paris-Saclay, CEA, CNRS, 18 Route du Panorama, 92265 Fontenay-aux-Roses, France; 2 Molecular Imaging Research Center, CEA, 18 Route du Panorama, 92265 Fontenay-aux-Roses, France; 3 Paris Brain Institute, Alzheimer’s and Prion Diseases Team, CNRS, UMR 7225, INSERM 1127, Sorbonne University UM75, Paris, France; 4 Laboratoire Neuropathologie Raymond Escourolle, Pitié, APHP, Salpetriere Hospital, Sorbonne University, 47, Blvd l’Hopital, 75651 Paris Cedex 13, Paris, France; 5 Neuro-CEB Neuropathology Network Network: Plate-Forme de Ressources Biologiques, Bâtiment Roger Baillet, Hôpital de la Pitié-Salpêtrière, 47-83 boulevard de l’Hôpital, 75651 Paris Cedex 13, France

**Keywords:** β-amyloid pathology, Alzheimer’s disease, Aβ transmission

## Abstract

In humans, iatrogenic transmission of cerebral amyloid-β (Aβ)-amyloidosis is suspected following inoculation of pituitary-derived hormones or dural grafts presumably contaminated with Aβ proteins as well as after cerebral surgeries. Experimentally, intracerebral inoculation of brain homogenate extracts containing misfolded Aβ can seed Aβ deposition in transgenic mouse models of amyloidosis or in non-human primates. The transmission of cerebral Aβ is governed by the host and by the inoculated samples. It is critical to better characterize the propensities of different hosts to develop Aβ deposition after contamination by an Aβ-positive sample as well as to better assess which biological samples can transmit this lesion. Aβ precursor protein (huAPP_wt_) mice express humanized non-mutated forms of Aβ precursor protein and do not spontaneously develop Aβ or amyloid deposits. We found that inoculation of Aβ-positive brain extracts from Alzheimer patients in these mice leads to a sparse Aβ deposition close to the alveus 18 months post-inoculation. However, it does not induce cortical or hippocampal Aβ deposition. Secondary inoculation of apparently amyloid deposit-free hippocampal extracts from these huAPP_wt_ mice to APP_swe_/PS1_dE9_ mouse models of amyloidosis enhanced Aβ deposition in the alveus 9 months post-inoculation. This suggests that Aβ seeds issued from human brain samples can persist in furtive forms in brain tissues while maintaining their ability to foster Aβ deposition in receptive hosts that overexpress endogenous Aβ. This work emphasizes the need for high-level preventive measures, especially in the context of neurosurgery, to prevent the risk of iatrogenic transmission of Aβ lesions from samples with sparse amyloid markers.

## Introduction

Epidemiological data suggest that, in humans, iatrogenic cerebral Aβ-amyloidosis can be induced following administration of cadaver-sourced human growth hormone [3, 9] or dura mater graft [8] containing amyloid-β (Aβ) proteins as well as after cerebral surgeries potentially involving tools contaminated with Aβ [10]. In addition to their occurrence in parenchymal tissue, these iatrogenic induced lesions can affect cerebral vasculature leading to amyloid angiopathy sometimes associated to cerebral hemorrhages inducing dramatic clinical signs and fatality [1, 8, 10]. Aβ deposition can be induced experimentally in mouse models that overexpress mutated forms of Aβ protein precursor (AβPP) after intracerebral inoculation of Aβ-containing brain extracts issued from transgenic mouse models of amyloidosis or from Alzheimer’s disease patients [5, 14]. The experimental transmission of Aβ-amyloidosis is considered to be related to Aβ seeds that act as self-propagating agents responsible for its initiation, progression and spreading in the brain. It has been observed that there is variability in the development of Aβ deposition between different hosts, e.g. different mouse models, which implies that host factors are critical for in vivo seeding [14]. The ability to induce Aβ deposition is also governed by the inoculated samples [14]. In order to assess the risk of iatrogenic contamination, it is critical: (1) to extensively characterize the hosts in which Aβ deposition can be induced, especially in animals with low propensities to develop amyloidosis; (2) to evaluate the potential of different brain samples to induce Aβ deposition. Our group recently showed that inoculation of human AD brain extracts to non-human primates that have a cerebral environment close to the human one can induce cerebral Aβ deposition [5]. Here, we found that intra-hippocampal inoculation of human AD brain extracts to huAPP_wt_ mice, a model that expresses humanized non-mutated forms of AβPP and does not spontaneously develop amyloid deposits, induces slight Aβ deposition in regions surrounding the alveus but not in other parts of the hippocampus or brain regions. This suggests that induction of cerebral Aβ deposition is low in models that have a low propensity to develop amyloid pathology. We then showed that apparently Aβ-deposit-free hippocampal samples from AD-inoculated huAPP_wt_ mice enhance Aβ deposition in the APP_swe_/PS1_dE9_ mouse model 9 months after their intrahippocampal inoculation. Thus, Aβ seeds can be transmitted from apparently Aβ-deposit-negative samples and induce Aβ deposition in hosts that have a high propensity to develop amyloid pathology. This suggests that the prevention of iatrogenic amyloid transmission from one patient to another does not rely solely on the amyloid status of the donors since samples with sparse Aβ lesions can induce pathology in a receptive host.

## Materials and methods

### Human brain samples

Frozen brain tissue samples (parietal cortex) from two Alzheimer’s disease patients (Braak stage VI, Thal phases 5 and 4, respectively) and one control individual that did not show clinical or histological signs of neurological disease (Braak stage/Thal phase 0) were obtained through a brain donation program of the Brainbank Neuro-CEB Neuropathology Network. The consent forms were signed by either the patients themselves or their next of kin in their name, in accordance with French bioethics laws. The Brainbank Neuro-CEB Neuropathology Network has been declared at the Ministry of Higher Education and Research and has received approval to distribute samples (agreement AC-2013-1887).

Detailed histological and biochemical characterization of these brains were previously published, as well as the preparation and assessment of human brain homogenates [5]. Operators were blinded to the status of the patients. The routine detection of Aβ and tau deposits was performed with the 6F3D anti-Aβ antibody (Dako, Glostrup, Denmark, 1/200) and polyclonal anti-tau antibody (Dako, Glostrup, Denmark, 1/500). Parietal cortex homogenates (20% weight/volume in a sterile 5% glucose solution) were aliquoted into sterile polypropylene tubes and stored at − 80 °C until use. The 20% aliquoted homogenates were diluted to 10% (w/v) in sterile Dulbecco’s phosphate-buffered saline (PBS, Gibco, ThermoFisher Scientific, France) extemporaneously prior to inoculation in mice. The extracts from the two Alzheimer brain samples were shown to be able to induce amyoid deposition four months after inoculation in two-month-old female APP_swe_/PS1_dE9_ mice [5].

### Ethical statement for animal experiments

All animal experiments were conducted in accordance with the European Community Council Directive 2010/63/ECC. Animal care was in accordance with institutional guidelines and experimental procedures were approved by local ethical committees (APAFIS 2015062412105538 v1; ethics committees CEtEA-CEA DSV IdF N°44, France). They were performed in a facility authorized by local authorities (authorization #D92-032-02), in strict accordance with recommendations of the European Union (2010-63/EEC), and in compliance with the 3R recommendations. Animal care was supervised by a dedicated veterinarian and animal technicians. Animals were housed under standard environmental conditions (12-h light-dark cycle, temperature: 22 ± 1 °C and humidity: 50%) with ad libitum access to food and water.

### Animals and overall experimental plan

#### Transgenic mouse models

**Fig. 1:**
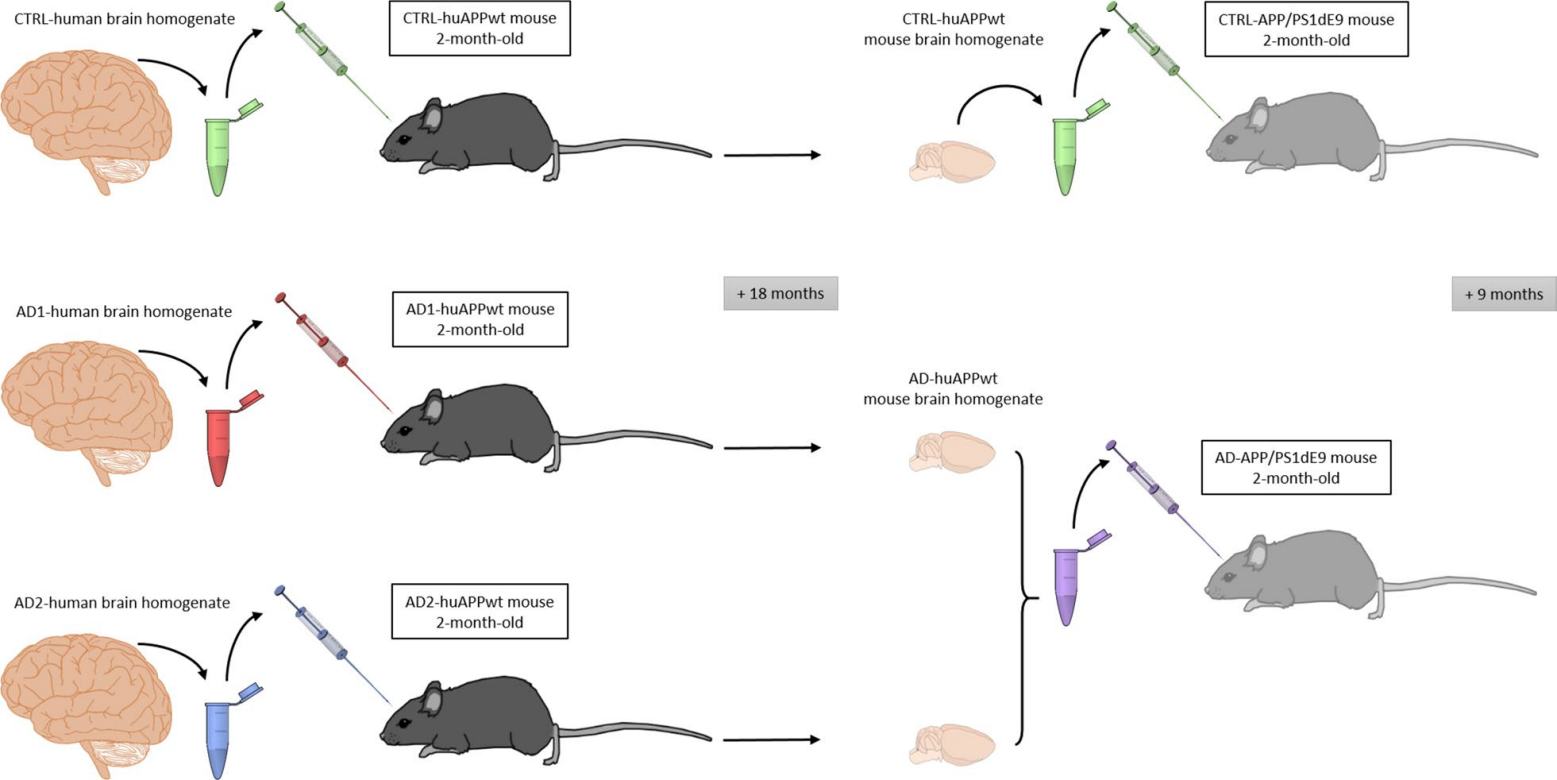
Experimental scheme summarizing the injection procedures and time points

The overall experimental plan is described in Fig. 1.

First, three experimental groups were initially created for experiments involving female huAPP_wt_ mice. These transgenic animals express a wild type form of the β-amyloid peptide precursor (AβPP) [16]. Mice involved in this study were heterozygous. These mice do not display any Aβ deposits even at late age [15]. HuAPP_wt_ mice were stereotaxically inoculated, in the hippocampus, at 2 months of age with AD brain homogenates from the two different AD patients (AD-huAPP_wt_, n = 6 for AD1, n = 6 for AD2) or with control human brain homogenate (CTRL-huAPP_wt_, n = 8). Their brains were collected at 20 months of age (18 months post-inoculation) and used for histological and biochemical analyses. Data from huAPP_wt_ mice inoculated with AD brain homogenates were pooled for statistical analysis. We thus compared two groups of animals, CTRL-huAPP_wt_ (n = 8) and AD-huAPP_wt_ (n = 12). Brains from huAPP_wt_ mice were also used to prepare hippocampal homogenates for secondary injection into APP_swe_/PS1_dE9_ mice. Two homogenates were prepared. The first one was made from the hippocampi of huAPP_wt_ mice inoculated with either AD1 (n = 6) or AD2 (n = 6) that were pooled to serve as a single homogenate. The second one was issued from the hippocampi of huAPP_wt_ mice inoculated with CTRL brains (n = 8). The APP_swe_/PS1_dE9_ transgenic mice express mutated forms of both human AβPP (APP_swe_: KM670/671NL) and presenilin 1 (deletion of exon 9) at high levels all throughout the brain and present with amyloid plaques and amyloid angiopathy [4]. Two experimental groups were followed-up: APP_swe_/PS1_dE9_ mice inoculated with brain homogenates from AD-huAPP_wt_ mice (AD-APP_swe_/PS1_dE9_, n = 6) or from CTRL-huAPP_wt_ mice (CTRL-APP_swe_/PS1_dE9_, n = 5). The mice were stereotaxically injected, in the hippocampus, at 2 months of age. Their brains were collected at 11 months of age (9 months post-inoculation).

#### Stereotaxic injections and mouse brain collection

Inoculations were performed bilaterally in the dorsal hippocampus (AP − 2 mm, DV − 2 mm, L ± 1 mm [17]). The animals were anaesthetized by an intraperitoneal ketamine-xylazine injection (Imalgène 1000, Merial, France (1 mg/10 g); 2% Rompun, Bayer Healthcare, Leverkusen, Germany (0.1 mg/10 g)) and placed in a stereotaxic frame (Phymep, France). Respiration rate was monitored and body temperature was maintained at 37 ± 0.5 °C with a heating blanket during surgery. After making a midline incision of the scalp, burr holes were drilled in the appropriate location. Bilateral intrahippocampal injections of 2 µL 10% brain homogenates were performed with a 26-gauge needle. The surgical area was cleaned before and after surgery (iodinate povidone, Vetedine, Vetoquinol, France), the incision sutured, and the animals placed in an incubator (temperature 25 °C) and monitored until recovery from anesthesia.

Animals were euthanized with an overdose of sodium pentobarbital (100 mg/kg intraperitoneally), followed by intracardiac perfusion with phosphate-buffered saline (PBS, Gibco, ThermoFisher Scientific, France) for APP_swe_/PS1_dE9_ mice only. Indeed, to preserve soluble Aβ species at best in the brain of huAPP_wt_ mice initially inoculated with human brain homogenates, we decided not to drain their brains with PBS. The left hemisphere was post-fixed with 4% paraformaldehyde in PBS for histological analysis. The right hemisphere was dissected to extract the hippocampus, which was immediately snap-frozen in liquid nitrogen and stored at − 80 °C for biochemical analysis and homogenate preparation.

#### huAPPwt mouse brain homogenate preparation

Hippocampi from huAPP_wt_ mice were sonicated 6 times (cycle 0.5, amplitude 30%, Heidolph, Entraigues sur la Sorgue, France) in Dulbecco’s PBS (10% m/m). They were then homogenized using ceramic beads (CK14-KT03961-1-003.2) and a Precellys 24 tissue homogenizer (Bertin Instrument, France) at 5000 rpm for 20 s. Samples were vortexed for 2 min, sonicated for 5 s (cycle 1, 40 amplitude units, 80 W) and centrifugated at 3000 *g* for 5 min. The supernatant was aliquoted and stored at − 80 °C. Samples were extemporaneously sonicated 20 times (cycle 0.5, 20 amplitude units) before injection in APP_swe_/PS1_dE9_ mice. A fraction of all the huAPP_wt_ mouse hippocampal homogenates, either AD- or CTRL-inoculated, were pooled to prepare the two homogenates injected in the brains of APP_swe_/PS1_dE9_ mice.

### Immunohistochemistry and microscopic image analysis

The 4% paraformaldehyde post-fixed hemispheres were cryoprotected using 15% and 30% sucrose solutions. Series of brain coronal sections (40-µm-thick) were cut on a sliding freezing microtome (SM2400, Leica Microsytem). The floating histological serial sections were preserved in a storage solution (30% glycerol, 30% ethylene glycol, 30% distilled water, and 10% phosphate buffer) at − 20 °C until use. Serial sections of the entire brain were stained for the evaluation of Aβ pathology (4G8 immunohistochemistry and Congo red staining). For 4G8 immunohistochemistry brain sections were rinsed with PBS, pre-treated with 70% formic acid for 3 min (only for biotinylated-4G8) and then incubated in 0.3% hydrogen peroxide for 20 min. Sections were then blocked with PBS-0.2% Triton (Triton X–100, Sigma, St Louis, MO, USA) and 4.5% normal goat serum (NGS) for 30 min before overnight incubation with biotinylated-4G8 at 4 °C (1:500; Biolegend Covance #SIGNET-39240, monoclonal). The sections stained were rinsed with PBS and then incubated with ABC Vectastain (Vector Labs) before diaminobenzidine tetrahydrochloride (DAB) revelation (DAB SK4100 kit, Vector Labs). For Congo red staining, sections were pretreated with 1% NaOH in 80% EthOH saturated with NaCl for 30 min. Then, they were immersed in the same solution saturated with Congo red for 30 min. Image of stained sections were digitized with a Zeiss Axio Scan.Z1 (Zeiss, Jena, Germany) whole slide imaging microscope at X20 (0.22 µm in plane resolution). Sections stained for Aβ or Congo red were blindly analyzed using ImageJ software [18]. Aβ deposits were segmented from 4G8-immunostained sections by applying a threshold (value of 100) on the blue component of the 8-bit images. Aβ burden was evaluated as the percentage of surface occupied by the 4G8 staining inside delineated regions of interest as well as the number of plaques per unit of surface. They were quantified in four regions: the hippocampus, parietal cortex, entorhinal cortex, and a region that follows the virtual ventricle that borders the alveus of the hippocampus towards the lateral ventricle. In the hippocampus, ROI definition was based on manual drawing following the region shape. In the parietal and entorhinal cortices, it relied on circles of constant diameter. For the alveus, ROIs were ribbons of 88 µm wide centered on the alveus (one per section, all labelled sections per mouse, selection brush tool from ImageJ). Four to seven sections were used for each animal depending on the number of available sections. Scientists who performed the analyses were blinded to the inoculation groups. Evaluations from Congo-red stained sections used similar methods (but with a threshold of 90), based on the green component of the 8-bit images. As lesions were more discrete, ribbons used for alveus ROIs were 35 µm wide. Labelling from structure with a diameter inferior to 7 µm were excluded from the quantification to avoid measures of background signal.

### Quantification of Aβ by immunoassays

Hippocampal extracts from both mouse strains were homogenized in Dulbecco’s PBS (10% m/m) and sonicated 6 times (cycle 0.5, 30% amplitude). They were incubated with a lysis buffer at a final concentration of 50 mM Tris HCl pH7.4, 150 mM NaCl, 1% Triton X-100 supplemented with protease and phosphatase inhibitor cocktails, and sonicated again as previously described. Samples were centrifugated at 20,000 *g* for 20 min at + 4 °C, the supernatant was collected as the soluble fraction and stored at − 80 °C until use. Aβ was measured by an electrochemiluminescence (ECL)-linked immunoassay (Meso Scale Discovery, MSD). The MSD V-PLEX Aβ peptide panel 1 (6E10) kit was used according to the manufacturer’s instructions. Briefly, samples were diluted 10-fold or 25-fold in the provided dilution buffer, respectively for soluble and insoluble fractions. Meanwhile, 96-well plates pre-coated with capture antibodies against Aβx-40 and Aβx-42 were blocked for 1 h and washed three times according to the manufacturer instructions, at room temperature. The SULFO-TAG anti-Aβ 6E10 detection antibody solution was then added to the wells and co-incubated with the diluted samples or calibrators at room temperature with shaking for 2 h. After washing, MSD Read Buffer T was added to the wells and the plate was read immediately on a MSD Sector Imager 2400. Data were analyzed using the MSD DISCOVERY WORKBENCH software 4.0. Internal samples were used for quality control of the assay performance and inter-plate variability. All samples and calibrators were run in duplicates.

### Statistics

Statistical analysis was performed using GraphPad Prism software, version 8, using a Mann-Whitney test (one-tailed). Data are shown on scattered dot plots with median and interquartile range.

## Results

### Human Alzheimer’s disease brain extracts induce a slight amyloid pathology in huAPP_wt_ mice

We bilaterally inoculated brain extracts from clinically and pathologically confirmed AD patients into the hippocampus of huAPP_wt_ mice. A brain extract from a non-AD patient was used as control. In a previous study, we demonstrated that, 4 months after inoculation, APP_swe_/PS1_dE9_ mice inoculated with the same AD brain extracts display increased amyloid plaque deposition and higher level of biochemically detectable Aβ as compared to mice inoculated with a CTRL human brain extract [5].

**Fig. 2:**
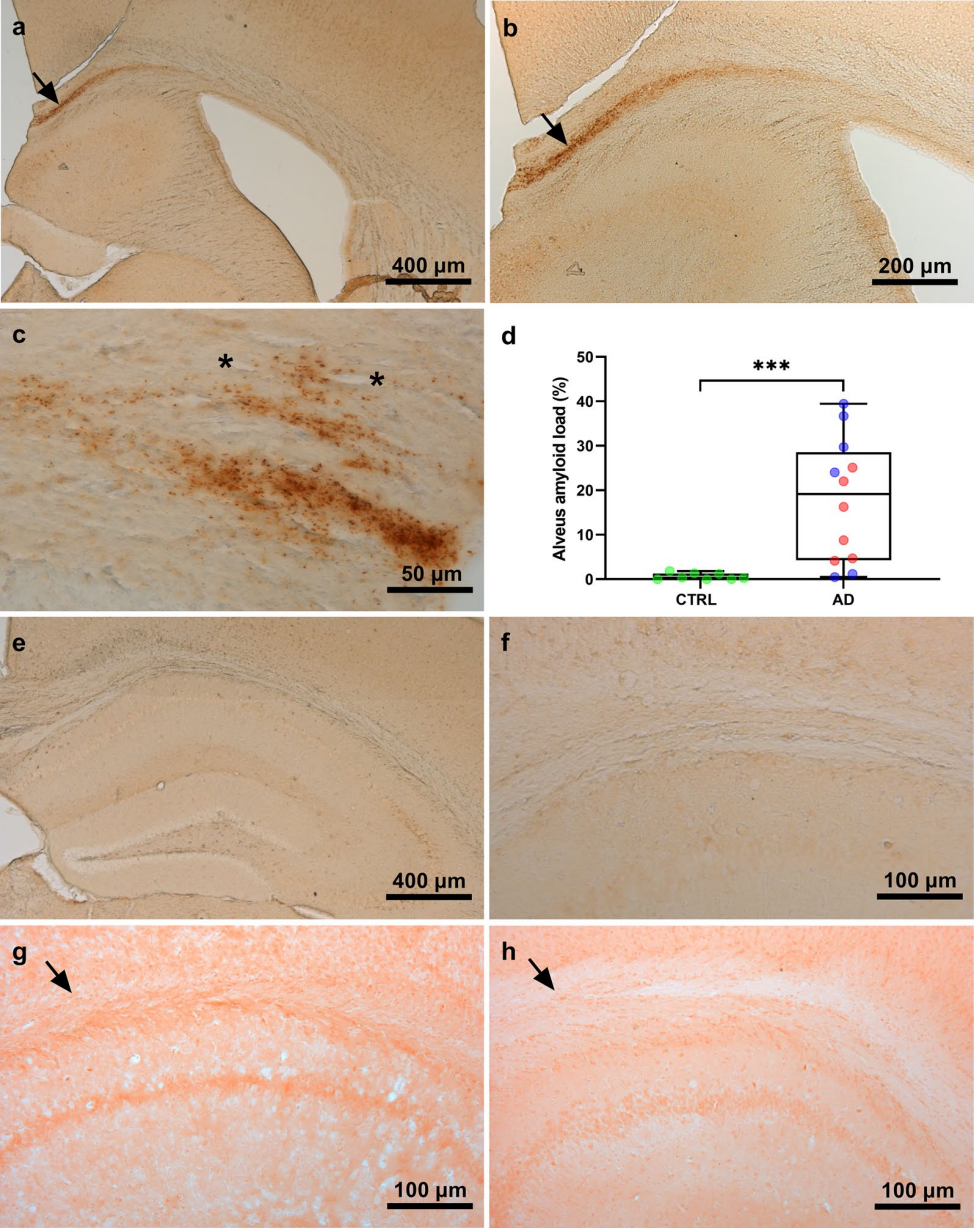
Detection of Aβ in the huAPP_wt_ mice inoculated with AD or CTRL brain. Mice inoculated with human brain homogenates were euthanized 18 months after the inoculation. Brain sections were stained with anti-Aβ (4G8-biotinylated) antibody. Staining was detected in the region surrounding the alveus of almost all the mice inoculated with the AD brains (**a**–**c**, arrows) but not the CTRL-brains (**e**–**f**). There was no staining in the hippocampus or cortex of the inoculated mice. 4G8 staining was not observed in the close vicinity of blood vessels (asterisk) as shown in a mouse inoculated with AD brain extract (**c**). Congo red did not stain any deposits in the inoculated mice, as shown in the alveus of an AD- (**g**) or CTRL-inoculated animal (**h**). 4G8-labeling was quantified in the alveus (**d**). First, regions of interest corresponding to the alveus were defined as ribbons of 88 µm wide centered on the virtual ventricle that borders the alveus of the hippocampus. Amyloid burden in this region was defined, using a thresholding method, as the percentage of surface occupied by the 4G8-staining inside the regions of interest. This analysis showed significant difference between CTRL and AD mice (U = 9; *p* = 0.001)

Using 4G8 biotinylated antibodies or Congo red, we did not detect any Aβ or amyloid deposits in the cortex or hippocampus of the huAPP_wt_ mice whether they were inoculated with human AD- or CTRL-brains. A clear labelling for Aβ was however detected in 4G8-stained sections in the region surrounding the alveus of almost all the mice inoculated with the AD brains (Fig. 2a–c). It consisted of diffuse extracellular deposits that did not adopt the morphology of plaques and were not present around blood vessels (Fig. 2c). This labelling was not detectable in the CTRL-inoculated mice (Fig. 2e–f). Congo-red stained sections did not reveal any amyloid deposits in AD- or CTRL-inoculated mice (Fig. 2g–h). Quantification of 4G8 sections confirmed the significantly higher Aβ load in AD-inoculated mice as compared to CTRL-inoculated mice (Fig. 2d, U = 9; *p* = 0.001) and only 2/12 AD-inoculated mice did not display obvious Aβ load. Biochemical analysis of huAPP_wt_ mouse hippocampus extracts demonstrated similar amounts of soluble Aβ_1-42_ (U = 26; p = 0.11) or Aβ_1-40_ (U = 32.50; *p* = 0.26) in both AD and CTRL groups (data not shown).

### Induction of amyloid pathology in APP_swe_/PS1_dE9_ mice after inoculation of brain samples from huAPP_wt_ mice primarily inoculated with AD brains

**Fig. 3:**
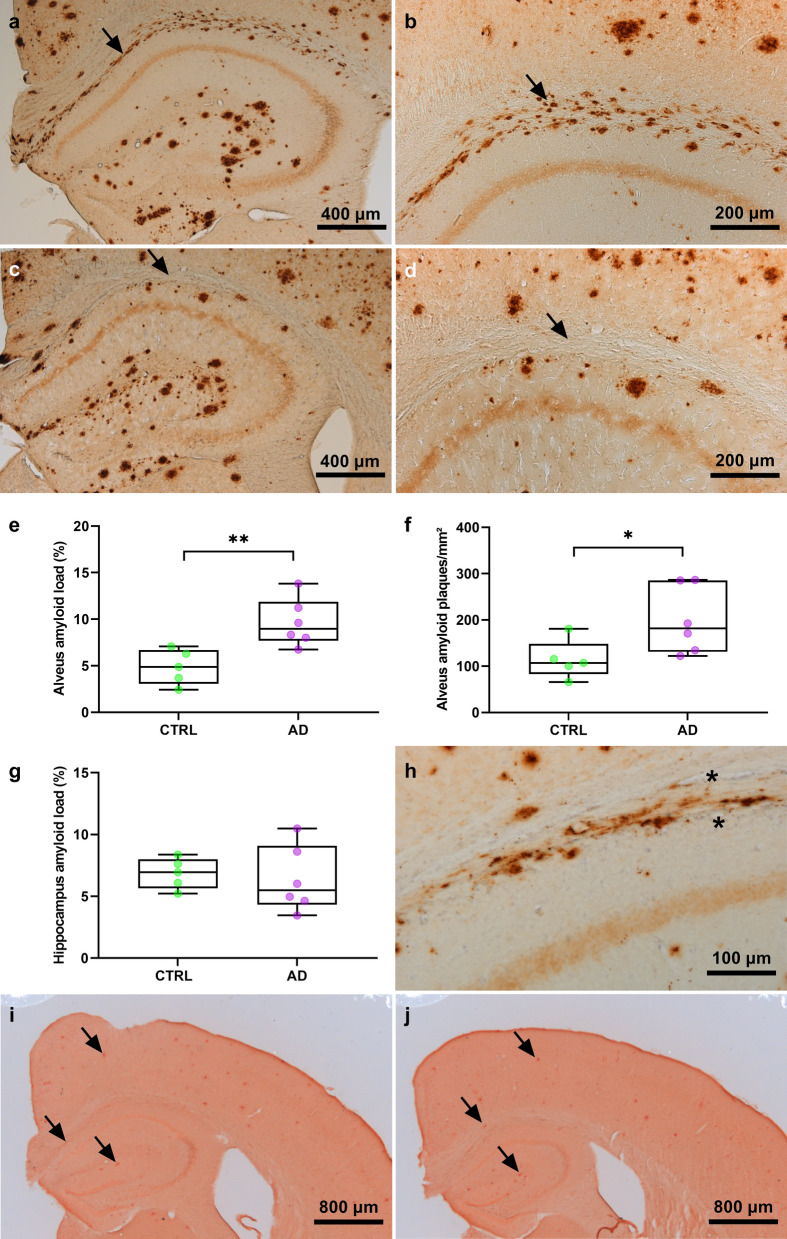
Increased Aβ deposition in the alveus of 11-month-old APP_swe_/PS1_dE9_ mice inoculated with AD-huAPP_wt_ brain homogenate. APP_swe_/PS1_dE9_ mice were inoculated with apparently amyloid deposit-free hippocampus extracts from huAPP_wt_ mice previously inoculated with human brain homogenates and euthanized 18 months after the inoculation. Aβ (biotinylated 4G8) stained brain sections from the APP_swe_/PS1_dE9_ mice showed plaques in the hippocampus of all mice (**a**–**d**). Aβ deposition was also seen in regions surrounding the alveus of the mice inoculated with AD-huAPP_wt_ brains (**a**–**b**, arrows). 4G8 staining did not involve blood vessels (asterisk) as highlighted here in a mouse inoculated with AD-huAPP_wt_ (**h**). 4G8-positive load (**e**) and the number of plaques per surface unit (**f**) were significantly increased in the region surrounding the alveus of AD-APP_swe_/PS1_dE9_ mice compared to the CTRL-APP_swe_/PS1_dE9_ (U = 1, *p* = 0.004 **, and U = 3, *p* = 0.015 *, respectively). **g** Amyloid load was not different in the hippocampus of AD-APPswe/PS1dE9 and CTRL-APPswe/PS1dE9 mice (U = 11, *p* = 0.3). Congo red stained brain sections from the APP_swe_/PS1_dE9_ mice showed amyloid plaques (arrows) in the hippocampus and cortex of mice from AD (**i**) and CTRL groups (**j**)

Hippocampal homogenates from AD-huAPP_wt_ or CTRL-huAPP_wt_ mice were stereotaxically injected in the hippocampus of APP_swe_/PS1_dE9_ mice (AD-APP_swe_/PS1_dE9_ or CTRL-APP_swe_/PS1_dE9_). Nine months post-inoculation, as expected, 4G8-positive Aβ plaques were detected in the hippocampus and cortex of both AD-APP_swe_/PS1_dE9_ (Fig. 3a, b) and CTRL-APP_swe_/PS1_dE9_ (Fig. 3c, d) animals. A clear labelling for Aβ, in the form of plaques, was also detected in the region surrounding the alveus of almost all the AD-APP_swe_/PS1_dE9_ mice, and to a lower extent of CTRL-APP_swe_/PS1_dE9_ mice. The morphology of the Aβ deposits observed in the alveus differed in the APP_swe_/PS1_dE9_ compared to that seen in the huAPP_wt_ mice. In the APP_swe_/PS1_dE9_ Aβ formed plaques while it was more diffuse in AD-huAPP_wt_ mice (Fig. 2a, b). However, as for AD-huAPP_wt_ mice, Aβ was not deposited within blood vessels of the APP_swe_/PS1_dE9_ mice (Fig. 3h). Quantification of 4G8-stained histological sections revealed an increased Aβ load (Fig. 3e, Mann-Whitney test, U = 1, *p* = 0.004) and an increase in the number of Aβ plaques per surface unit (Fig. 3f, Mann-Whitney test, U = 3, *p* = 0.015) in the regions surrounding the alveus of the AD-APP_swe_/PS1_dE9_ mice as compared to CTRL-APP_swe_/PS1_dE9_ mice. These plaques were not different in size (U = 10, *p* = 0.2). There was not a statistically significant difference in Aβ load in the hippocampus (Fig. 3g, U = 11, *p* = 0.3), parietal cortex (not shown, U = 14, *p* = 0.5) or entorhinal cortex (not shown, U = 13, *p* = 0.4) of these two groups. The number of Aβ plaques per surface unit was also not different between these two groups in these regions (not shown, U > 10, *p* > 0.2). Staining with Congo red detected amyloid plaques in the hippocampus and cortex of AD-APP_swe_/PS1_dE9_ animals (Fig. 3i) or CTRL-APP_swe_/PS1_dE9_ animals (Fig. 3j). Some amyloid plaques were also detected in the region surrounding the alveus of almost all the mice. Whatever the regions, congophilic plaques were however less numerous and visible than 4G8-labelled plaques. Unlike for 4G8 staining, quantification of Congo red stained sections did not show differences between the two groups of APP_swe_/PS1_dE9_ animals either in regions surrounding the alveus (U = 10, *p* = 0.2) or in other regions as the hippocampus (U = 9, *p* = 0.2) or parietal cortex (U = 13, *p* = 0.4). Soluble Aβ_1-42_ and Aβ_1-40_ were assayed in the hippocampus of the APP_swe_/PS1_dE9_ animals inoculated with AD- or CTRL-huAPP_wt_ mouse brain homogenates. No differences were found between the two groups of animals (not shown, Aβ_1-42_: U = 11, *p* = 0.3; Aβ_1-40_: U = 12; *p* = 0.3).

## Discussion

Human AD-brain extracts were inoculated in the hippocampus of huAPP_wt_ mice that express human wild-type AβPP gene. We showed that, 18 months after inoculation, there was no induction of cortical or hippocampal Aβ deposits. However, extracts from the apparently Aβ-deposit-free hippocampus of these AD-inoculated animals induced Aβ-deposition in APP_swe_/PS1_dE9_ mice 9 months after inoculation. This suggests that Aβ seeds, able to foster Aβ deposition, were present in the hippocampus of huAPP_wt_ without being easily detectable.

Several studies have shown that the inoculation of human brains containing Aβ seeds can accelerate cerebral amyloid plaque occurrence in mouse models or primates [5, 14]. The ability to induce cerebral Aβ-amyloidosis experimentally highly depends on the hosts and the inoculated samples [14]. Inoculation of human brains in hosts that overexpress AβPP (i.e. in specific transgenic mice) leads to an acceleration of Aβ plaque occurrence. In particular, in a previous study, we showed that inoculation to APP_swe_/PS1_dE9_ mouse models of amyloidosis of the human AD brain extracts used in the current study induced Aβ depositions 4 months after the inoculation [5]. Studies in primates that have a natural expression of AβPP (100% homology with the human gene) showed that sparse Aβ deposits can also be induced following inoculation of Aβ seeds [5]. A previous study in huAPP_wt_ mice that express the human wild-type AβPP gene, which never develop Aβ plaques spontaneously, showed sparse Aβ deposition in the cortex and hippocampus in 40% of the animals 9 months post inoculation, in 65% of the animals 15 months post inoculation and in 100% of the animals 19 months post inoculation [15]. In our hands, small diffuse Aβ deposits were detected in the region surrounding the alveus, but Congophilic amyloid plaques were not detected. The lower detection of Aβ deposits in our study as compared to the previous study [15] might be related to a different preparation of the human samples, and potentially different sonication procedures.

We wondered why Aβ accumulation occurred in regions surrounding the alveus. Several articles reported Aβ deposition in this region after intrahippocampal inoculation of human AD-brain homogenates [2, 5, 11, 14, 21], amyloid-positive mouse brains [7, 14, 20, 22] or synthetic Aβ species [19, 20]. The presence of abnormal protein deposits in this region was also reported after inoculation of prion homogenates in the thalamus/brain midline with a needle that crossed the parietal cortex, the lateral ventricle and the hippocampus [6]. It was suggested that the orientated disposition of white matter fibers in this area might provide an efficient path for the diffusion from the inoculation sites towards the lateral ventricles to clear pathological proteins [13].

One of the critical questions to prevent iatrogenic transmission of amyloid concerns the propensity of the donor to induce Aβ-deposits or amyloidosis. Studies in transgenic mouse models of Aβ-amyloidosis proved that amyloid plaque-positive brains from non-demented subjects are able to induce cerebral Aβ-amyloidosis [2] while amyloid plaque-negative human brain samples were not able to induce Aβ-amyloid pathology [2]. Here, we show that homogenates from hippocampi free of Aβ plaques can induce Aβ deposition when injected into APP_swe_/PS1_dE9_ mice. We hypothesize that the huAPP_wt_ hippocampi from mice previously inoculated with AD brain extracts probably contained very low amount of Aβ seeds. Indeed, even if Aβ-deposits were not detected in the hippocampus, they were detected in the alveus that juxtaposes the hippocampus.This small amount of Aβ did not lead to Aβ plaque occurrence or stronger Aβ detection by biochemical analysis of Aβ_1-40_ or Aβ_1-42_ in the hippocampi of huAPP_wt_ animals. However, these samples could induce an increase Aβ deposition in regions surrounding the alveus, 9 months post inoculation after their inoculation in the hippocampus of APP_swe_/PS1_dE9_ mice. Evidence for Aβ deposition was provided by the increase in Aβ burden as well as the increase in the number of Aβ-plaques. This clearly outlines that brains with minimal amount of Aβ are able to induce Aβ deposition. Furthermore, a recent study showed long-term resilience (180 days) of transmissible but barely detectable Aβ seeds in AβPP null mice previously inoculated with the brain of mouse models of amyloidosis [23]. The study was based on inoculation of Aβ-containing brain extracts issued from very old transgenic mice with high amyloid load in AβPP null mice that cannot produce Aβ proteins. These mice were analyzed up to 6 months after inoculation and only slight Aβ accumulation (pg/ml range) could be detected by ultra-sensitive techniques in their brains. When these brains were intracerebrally inoculated in 3-month-old APP23 mice (harboring the APP_swe_ (APP KM670/671NL) transgene), they accelerated Aβ-deposition as early as 4 months after their inoculation, thus suggesting the long resilience—at least, up to 6 months– of the contaminating forms of Aβ [23]. This means that exogenous Aβ seeds from transgenic mice can remain in the brain at levels below routine detection, retain their pathogenicity in AβPP null mice, i.e. in the absence of replication, and therefore have extreme longevity. Unlike this latter study, our experiment was based on the inoculation of human (and not mouse) brain extracts. We speculate that the pathology developed in the APP_swe_/PS1_dE9_ mice may reflect a dual process based on the resilience of initial seeds and/or on the nucleation of the Aβ from the huAPP_wt_ mice. Congophilic plaques were however not modulated by the inoculation of AD-huAPPwt brain extracts. In any case, this study shows that very small amounts of Aβ seeds presumably issued from the human brain samples are able to induce an Aβ pathology. Nonetheless, the limitations of some ELISA kits to detect very low amounts of Aβ peptide are pointed out by some authors. We cannot rule out that Aβ would have been detected with more sensitive tools as Simoa technology. Unfortunately, we did not have any sample left to test this hypothesis. Based on our data, it is reasonable to suggest that soluble forms of Aβ can act as seeds that are able to induce Aβ aggregation in recipient hosts. This is consistent with previous data showing that supernatant from full brain extracts have a strong ability to induce Aβ aggregation while containing only low level (0.05%) of the brain Aβ pool [12].

Aside from sporadic and genetic forms of AD, iatrogenic transmissions are “new” ways to transmit an AD-related amyloid pathology. These iatrogenic transmissions can be based on surgical procedures and/or inoculation of cadaver brain extracted hormones. Elucidating the risk factors is critical to organize prevention procedures. Here, we outline that even small amounts or undetectable Aβ seeds can induce Aβ deposits in a recipient host. Previous studies have recommended the systematic use of prion diseases preventive measures in neurosurgery and the exclusion of patients with AD from donor programs. Since, as suggested by our results, Aβ deposit-negative samples can also induce Aβ pathology, it may be useful to also recommend prudence with samples from donors who could present unsuspected Aβ deposits.
